# Estimation of Caries Treatment Needs in First Permanent Molars of Lithuanian 5–6-Year-Old Children, Based on Caries Lesion Activity Assessment

**DOI:** 10.3390/medicina56030105

**Published:** 2020-03-02

**Authors:** Vita Mačiulskienė, Jaunė Razmienė, Vilija Andruškevičienė, Eglė Bendoraitienė

**Affiliations:** 1 Department of Dental and Oral Pathology, Faculty of Odontology, Lithuanian University of Health Sciences, 50161 Kaunas, Lithuania; 2 Department of Preventive and Paediatric Dentistry, Faculty of Odontology, Lithuanian University of Health Sciences, 50106 Kaunas, Lithuania; jaune.razmiene@lsmuni.lt (J.R.); vilija.andruskeviciene@lsmuni.lt (V.A.); egle.bendoraitiene@lsmuni.lt (E.B.)

**Keywords:** caries prevalence, first permanent molars, children, active caries lesions, dental plaque levels, toothbrushing frequency

## Abstract

*Background and Objectives:* Early detection of dental caries lesions at active stages of development can facilitate their monitoring and reduce needs for restorative dental care. This study aimed to describe the prevalence and caries treatment needs in first permanent molars of pre-school children, based on a caries lesion activity assessment, and in relation to participants’ ages, dental plaque levels and toothbrushing habits. *Materials and Methods:* Large cross-sectional dental caries survey using multistage cluster sampling was conducted among Lithuanian 4–6-year-old children attending kindergartens. For the present study purpose, all individuals presenting erupted permanent molars were selected. Thus, only 5–6-year-olds (*n* = 453) took part in this study. They were examined for caries by one calibrated examiner using Nyvad clinical diagnostic criteria that differentiate between active and inactive caries lesions. Dental plaque was assessed by the Silness-Löe index, and parents’ reports about toothbrushing frequency were collected. *Results:* Overall, 41% of permanent molars were affected by caries; 6-year-olds had more caries lesions than 5-year-olds (*p* < 0.05). Mean number of decayed and filled surfaces (DF-S) of all participants was 1.79 (SD 2.93), half of lesions were noncavitated, more than one-third were cavitated and fillings comprised less than one surface per child. Majority of lesions were active; prevalence of inactive lesions (all noncavitated) was 1% and 6% in 5- and 6-year-olds, respectively. Prevalence of active lesions increased with age; it correlated with plaque levels and with toothbrushing frequency (<0.001). Likelihood to detect active lesions was up to nine times higher in teeth with abundant plaque (odds ratio (OR) 8.73; confidence interval (CI) 5.35–14.25), and up to seven times higher in individuals brushing teeth irregularly (OR 6.88; CI 2.21–21.41). *Conclusions:* The obtained data indicate high treatment needs in the erupted permanent molars of the Lithuanian pre-school population and imply that caries management should primarily focus on improved biofilm removal, accompanied with regular use of fluoridated toothpaste.

## 1. Introduction

Dental caries is known as a multifactorial, noncommunicable disease determined by biological, behavioral, psycho-social and environmental factors [1]. It expresses itself by the interchanging episodes of de- and remineralization of the tooth surface caused by metabolic activities in the dental biofilm [1]. The dynamic nature of dental caries is reflected clinically by the activity status of the lesions in response to the numerous chemical interactions between the dental tissues and the surrounding oral environment [2,3]. The balance between such interactions, and consequently, the progression of the disease, can be controlled successfully if the management strategies are based on biological knowledge of the process development. High response of the early, noncavitated lesions to nonoperative measures such as fluoride therapy and control of the dental biofilm [4,5] implies that the detection of such lesions at their active stage of development can reduce the needs for restorative care and, possibly, contribute to the maintenance of dental health throughout life. Implementation of caries preventive approaches from the early stages of life has proved not only to be related with significant reduction of the disease prevalence and severity [6] but also to have a long-lasting effect [7].

The necessity to include noncavitated caries lesions in the measurements of the disease extent was recognized long ago [8,9] and gradually became a common modality in a number of epidemiological surveys of today. However, this is not sufficient when the aim is to estimate the actual treatment needs in populations. The priority should be given for clinical caries diagnostic criteria that assess the activity status of the detected lesions, since these criteria favor the best clinical practices directed towards nonoperative interventions [10,11]. The need for caries lesion activity assessment is recognized internationally [11]; however, the only available report from Lithuania about the lesion activity-based assessment of caries treatment needs addressed in the population of 12-year-old children [8].

In view of the above considerations, the aim of the present study was to assess the actual treatment needs of dental caries in the newly erupted first permanent molars of the pre-school Lithuanian children, based on the estimates of the lesion activity and, in relation to age, the dental plaque levels and the toothbrushing habits.

## 2. Materials and Methods

### 2.1. Study Design, Population and Sample Selection

The present analysis was derived from a large cross-sectional dental caries survey conducted among the Lithuanian 4–6-year-old children attending pre-school educational institutions (further, kindergartens) [12]. According to the Official Statistical Portal data, there are about 60,000 children of this age in Lithuania, and about 38,804 of them attend kindergartens.

The survey was performed during the period from March 2011 to May 2012. The research protocol was approved by the Kaunas Regional Committee of Ethics for Biomedical Research, Kaunas, Lithuania (Protocol No. BE-2-19, issue date 04.11.2009), following the permission of the State Data Protection Inspectorate (Protocol No. 2R-732). The written informed consent was obtained from all parents of the participating children, as well as from the leading persons of all educational institutions visited.

**Figure 1:**
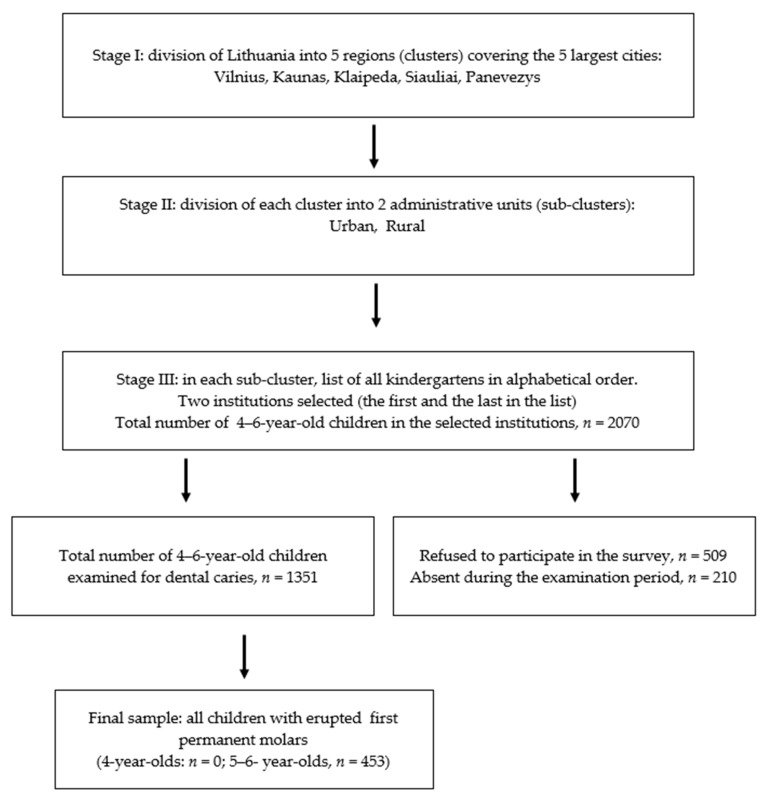
Flow-chart diagram of the study sample formation following multistage cluster sampling.

A method of multistage sampling was used for identification of the subjects for the survey (Figure 1).

The inclusion criteria for participation in the present study were (i) willingness to participate and (ii) presence of at least one permanent molar erupted. Thus, the final study group comprised 453 5–6-year-old participants.

### 2.2. Clinical Examinations

Clinical oral examinations were carried out in the kindergartens under standardized conditions, using a portable dental unit and the convertible chairs for the patients and for the examiners. The dental unit was equipped with a fiber optic light source, compressed air and a suction device. The evaluation of the dental status was performed using a standard explorer and a dental mirror. The clinical examinations were performed by one examiner (J.R.) who, prior to the study, had been extensively calibrated by one of the co-authors (V.M.) who had considerable experience with the clinical assessment of caries lesions, with respect to their activity and severity status (Nyvad criteria). For inter-examiner reliability testing, 30 children were examined repeatedly by two examiners (J.R. and V.M.), with an interval of 1 week between two examinations.

Dental caries was recorded using the clinical criteria described by Nyvad and co-authors [13]. Briefly, every surface of the erupted permanent molars was evaluated according to one of the following criteria:

0. sound surface,
1. active noncavitated lesion,
2. active enamel microcavity,
3. active enamel and dentin cavity,
4. inactive noncavitated lesion,
5. inactive enamel microcavity,
6. inactive enamel and dentin cavity,
7. filling,
8. filling + active lesion (noncavitated or cavitated),
9. filling + inactive lesion (noncavitated or cavitated),
10. tooth extracted due to caries.

Distinction between active and inactive lesions was made on the basis of a combination of visual (whitish, opaque versus discolored, shiny) and tactile (rough/leathery/soft versus hard/smooth) criteria. The inter-observer and intra-observer reliability for different lesion categories was almost perfect, as measured by weighted kappa (mean κ = 0.87, confidence interval (CI) = 0.82–0.91). 

The evaluation of oral hygiene status was based on the visual plaque assessment following Silness-Löe index [14]. The examination was performed on the buccal surfaces by running across the tooth surface with a probe, and the following scores were given to each surface:

11. no plaque,
12. film of plaque adhering to the free gingival margin and adjacent area of the tooth,
13. moderate accumulation of soft deposits on the tooth surface and gingival margin, which could be seen with the naked eye, and
14. plaque covering entire surface of the tooth.

### 2.3. Collection of Nonclinical Data

The parents of all children participating in the survey were asked to report about the frequency of toothbrushing at home. The reports were summarized as follows:

1. regular toothbrushing, 2 times per day,
2. regular toothbrushing, once per day,
3. irregular toothbrushing (several times per week), and
4. no toothbrushing at all.

No information about the type of toothpaste used was collected. However, most of the toothpaste available on the market in Lithuania contains fluoride in concentrations ranging from 250 to 1450 ppm. The concentration of fluoride in drinking water was very low in most regions of the country, varying between 0.1–0.3 mg/L, except two water supplies (in Klaipeda and Siauliai Districts), where it was measured to be 1.4 and 0.7 mg/L, respectively. The measurements were made in the national public health surveillance laboratory using the water samples collected at all sites of the survey.

### 2.4. Statistical Analysis

Analysis of the collected data was performed using the SPSS program (Statistical Package for Social Science for Windows, version 22.0, Chicago, IL, USA). The Kolmogorov-Smirnov test was used to test the normality distributions. When the obtained data did not meet the distribution normality condition, the significance level was verified by nonparametric Mann-Whitney U test. When carrying out the descriptive data analysis, the quantitative variables were expressed as means, M (with standard deviations, SD), and medians (with 25%–75% ranges, IQR). The interdependence of qualitative characteristics was evaluated using chi-square (χ^2^) criterion. The difference was considered to be statistically significant at the level of significance *p* < 0.05. Associations between the prevalence of active caries lesions and the recorded levels of dental plaque, as well as the reported toothbrushing frequency, were addressed by means of logistic regression analysis.

For the purpose of comparing the study results obtained using Nyvad criteria with those that would have been obtained using the World Health Organization (WHO) criteria [14], the Nyvad codes were converted as follows: 0;1;4 (Nyvad)—sound (WHO); 2;3;5;6;8 (Nyvad)—caries (WHO); 7;9 (Nyvad)—filled (WHO). For the odds ratio estimation, the dental plaque levels were dichotomized into two categories: no plaque (0+1) and plaque present (2+3). The reported toothbrushing habits were dichotomized into two categories as well: 1–2 times per day and irregularly/never. The predictors of high caries experience (expressed as a mean number of decayed and filled surfaces (DF-S)) in the permanent dentition, were addressed by means of a multivariate binary logistic regression analysis by adjusting for age, caries experience in the primary dentition (mean number of decayed, missing and filled surfaces, dmfs) and self-reported toothbrushing habits as independent variables. Spearman’s rank correlation coefficient (r) was calculated to assess the relationship between the caries experiences in the primary and in the permanent dentition.

## 3. Results

**Figure 2:**
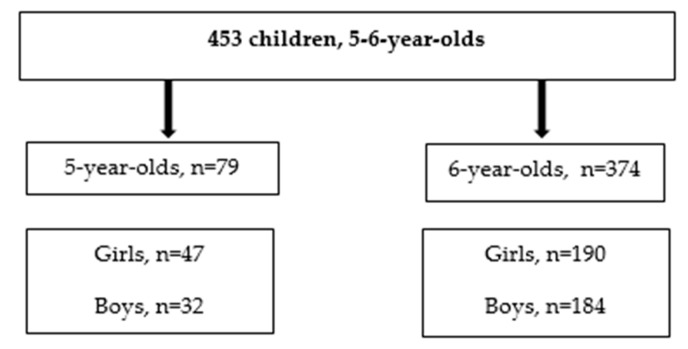
Distribution of study participants according to age and gender.

The distribution of the study participants according to their age and gender is shown in Figure 2.

All 453 5–6-year-old participants had at least one permanent molar erupted. The total number of the permanent molars under the investigation was 1531 (221 and 1310, in 5- and 6-year-olds, respectively). No extracted permanent molars were recorded; thus, the caries experiences in the group were evaluated based on the DF-S index.

**Figure 3:**
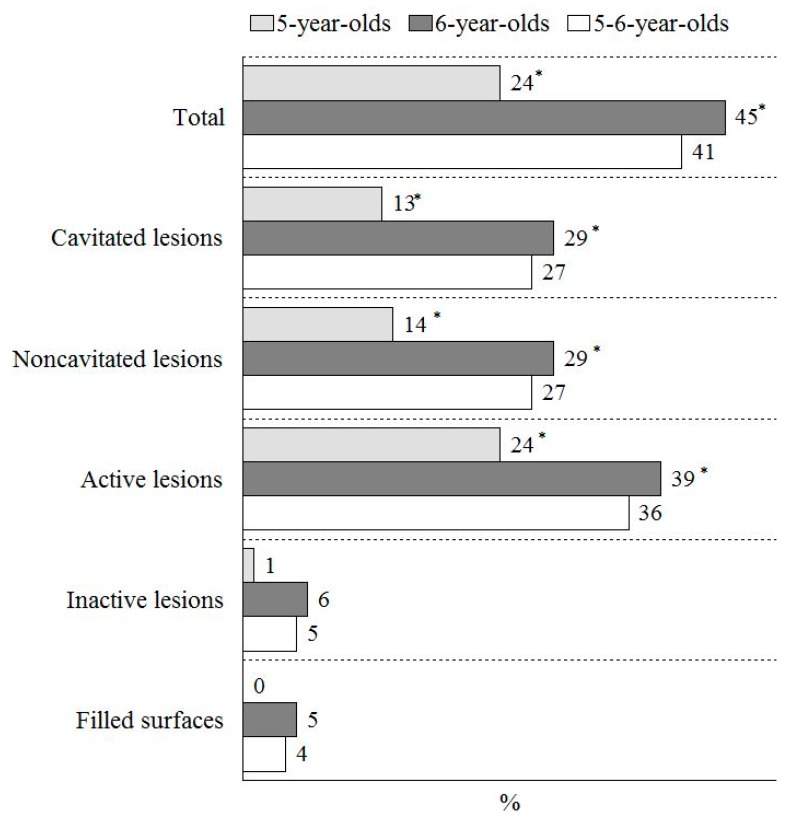
Prevalence of dental caries in the first permanent molars among 5- and 6-year-old children, with respect to lesion severity and activity by the Nyvad criteria. * *p* < 0.01, with respect to age group (χ^2^ test).

Figure 3 shows that 41% of all study participants had at least one caries lesion or a dental filling recorded in the permanent molars. The caries experiences in the permanent molars comprised mostly untreated caries lesions; only 4% of all participants were recorded as having fillings, and nearly no filled dental surfaces were recorded among the 5-year-olds. In the study groups, the noncavitated lesions were as prevalent as the cavitated lesions. The prevalence of dental caries (the noncavitated, as well as the cavitated lesions) in the first permanent molars was about two times higher among the 6-year-olds than among the 5-year-olds (Figure 3).

No difference in the caries prevalence in the first permanent molars was determined with respect to gender: 42% among the girls and 41% among the boys, respectively.

Majority of the caries lesions recorded in the permanent molars of the Lithuanian 5–6-year-olds were active, while the prevalence of inactive lesions was as low as 5% (Figure 3). All recorded inactive lesions were in the noncavitated stage of development. However, the prevalence of active noncavitated and cavitated lesions in the entire group of participants was similar: 22%, and 26%, respectively.

There was statistically significant differences in the prevalence of active caries lesions, with respect to age: 24% of the 5-year-olds had active caries, while 39% of 6-year-olds were recorded as having active caries lesions (Figure 3). There was more than two-fold difference between the mean DF-S values in the first permanent molars, based on the Nyvad criteria (DF-S-Nyvad), and when noncavitated lesions had been excluded from the estimation (DF-S-WHO) (Table 1). The mean caries experience was two times higher among the 6-year-olds than among the 5-year-olds (Table 1). The caries profile mostly comprised the untreated active caries lesions, and more than two-thirds of them were cavitated (Table 1). The mean number of active cavitated caries lesions was two times higher among the 6-year-olds than among the 5-year-olds (*p* = 0.01) (Table 1).

However, the mean values of the inactive caries lesions in the first permanent molars increased about four times when compared between the 5- and the 6-year-old children. 

Majority of all caries lesions recorded in the study group were located in the occlusal surfaces of the first permanent molars. The caries profile of the occlusal surfaces with respect to age is shown in Table 2. 

The difference in the mean number of active caries lesions in the first permanent molars of 5- and 6-year-old children remained statisttically significant when the estimations were adjusted for the differences in sample size (by randomly selecting the adjusted sample, *n* = 92, of 6-year-olds).

The distribution of the plaque scores was similar among the two age groups: (12% + 37%) and (10% + 41%) for scores (0+1) and (40% + 11%) and (39% + 11%) for scores (2+3) in 5-year-old and in 6-year-old children, respectively.

The toothbrushing habits were described based on the reports obtained from the parents of all 5–6-year-old participants. Routine daily toothbrushing was reported by 360 (80%) of all respondents: 145 (32%)—2 times and 215 (48%)—once per day, respectively. The rest (20%) of the study group (90 individuals) brushed their teeth irregularly, and three (0.7%) individuals did not brush their teeth.

Analysis of the prevalence of active caries lesions in the first permanent molars, with respect to the presence of dental plaque on the permanent molars, showed that it significantly correlated with the recorded plaque levels (r = 0.433; *p* < 0.001). The percentage of individuals presenting with active caries was significantly higher in the group of children scored with the plaque scores 2 and 3 (Table 3). There was a significant correlation with the self-reported toothbrushing frequency, indicating that the individuals who brushed their teeth 1–2 times per day had significantly fewer active caries lesions than those who did it less frequently (Table 3). Moreover, there was a statistically significant difference in the prevalence of active caries lesions among those individuals who brushed their teeth two times per day and those who did it less frequently (χ^2^ = 58.181; df = 2; *p* < 0.001).

Logistic regression analysis of the predictors of a high caries experience in permanent dentition showed that those children who had their caries levels in the upper tertile of the dmfs distribution were four times more likely to appear in the upper tertile of the DF-S distribution (Table 4). Furthermore, age of the participants, as well as their toothbrushing frequency, were positively associated with a high caries experience in the permanent teeth (Table 4).

## 4. Discussion

The results of the present study showed that the first permanent molars of Lithuanian 5–6-year-old kindergarten children were significantly affected by dental caries. Dental caries experiences differed in accordance with age, presenting twice as many lesions recorded among the 6-year-old children than among the 5-year-old children. Moreover, the prevalence of the cavitated caries lesions was more than two times higher among the 6-year-olds, indicating the likelihood of the fast development of the caries process in the newly erupted teeth. Active lesions dominated in both age groups. Interesting to note that, although the prevalence of inactive caries lesions was generally very low (5%), there was a 5-fold difference among the two age groups, reflecting a certain increase in inactive lesions amongst older children. 

Only a few reports about dental caries prevalence in the permanent dentition of 5–6-year-old children can be found in the literature. According to WHO, the youngest recommended age group for oral health surveys in permanent teeth is 12 years, while the five-years age is of interest only in relation to caries levels in primary dentition [15]. Although the caries prevalence markedly declined in many countries over the world and a significant proportion of 12-year-old children in a number of countries are still caries free, there are populations with a less pronounced reduction of the disease, mainly reflecting the disparities in socioeconomical backgrounds of coming from the less developed countries [16]. In such populations in particular, estimation of the disease prevalence and severity in permanent dentition should be performed much earlier, in order to record the caries lesions before they reached advanced stages of development.

The period of tooth eruption is considered a major challenge for caries development, due to the limited mechanical function and, consequently, facilitated accumulation of the dental biofilm on the erupting surfaces [17]. Moreover, the progression of caries lesions can be relatively fast in the newly erupted teeth. As an example, about 25% of all permanent molars in Finnish children were restored within a year of eruption [18]. The lesion progression in the occlusal surfaces can be even faster than in the other types of surfaces. Despite the overall caries decline, the occlusal surfaces remain the sites in the dentition which are most frequently attacked by dental caries [19]. The results of the present study support this observation: more than 70% of the total caries experienced in the study population comprised the lesions in the occlusal surfaces. Although part of the initially formed caries lesions may become arrested when teeth reach the occlusal plane, a significant proportion of these lesions remain active and in need of proper management [20]. Moreover, the patients with active caries lesions are regarded as disease-active and have a high risk of caries development, unless actions are taken to monitor this process [19]. As shown in the present survey, the prevalence of noncavitated caries lesions among the 6-year-olds was twice as high as among the 5-year-olds, and the majority of them were still active. In the present study, similar to the data obtained in the French population of the 6-year-old children [21], more than 30% of the study participants had either untreated or filled cavitated lesions in the first permanent molars. 

Presence of the undisturbed dental biofilm on a dental surface is known to be a major contributing factor to the development of dental caries.Thus, the prevalence of active caries lesions in the first permanent molars detected in the present study population correlated significantly with the recorded dental plaque levels; there was up to nine times higher a chance to detect active caries lesions in those children who had an abundant plaque present on the dental surfaces (the plaque scores 2 and 3). This is not a new issue; it has been shown in a number of earlier studies that caries lesion activity is related to the plaque scores on the dental surfaces [22,23,24,25]. Moreover, a significant correlation of the caries experienced in the newly erupted permanent teeth with the caries experienced in the primary dentition, as well as with the reported toothbrushing frequency of the participating children, was observed. It is well-known that past caries experiences are a strong indicator of caries risk in individuals [26]. However, dental caries is often called a behavioral disease, where individual factors such as dietary and oral hygiene habits play a role in the control of disease progression. Although the information about the oral hygiene habits reported by the parents of the participating children in this study had a subjective character, it was in line with the recently published results of the meta-analysis showing that individuals who state that they brush their teeth less frequently are at greater risk to develop new caries lesions than those who brush more frequently [27]. This implies that the strategy of caries management in the pre-school population should primarily focus on the improved biofilm removal, accompanied with a regular use of fluoridated toothpaste.

In this study, the visual dental plaque assessment was performed using the Silness–Löe index [14]. This index is based on the plaque accumulation estimates on the smooth surfaces of teeth and does not provide information about the status of the occlusal surfaces. One could consider it as a limitation of this study. However, the main argument for choosing the commonly used Silness–Löe index was related to the fact that dental plaque accumulation is always more pronounced on the smooth surfaces of teeth in contrast to the occlusal surfaces where it is continuously disturbed by the masticatory movements. Therefore, we believe that this index has a better capability to reflect the general oral hygiene level of the individuals than the occlusal indices.

Another important aspect pertaining to comparison of the disease prevalence/severity data in different populations is the utilization of different diagnostic classifications. Substantial variability in the clinical criteria for caries detection among the researchers can have an impact on the reports regarding the disease-affected and disease-free individuals and, consequently, lead to different estimations of treatment needs [11]. As an example, similarly to the present investigation, a survey among 7–8-year-old children was performed around the same time in Poland. Dental caries was recorded in 15%–17% of the study population [28]. This estimate is significantly lower than in the present population of the Lithuanian 5–6-year olds. However, the assessment of dental caries in the above-mentioned survey was based on the CAST (Caries Assessment Spectrum and Treatment) index, and all dental surfaces with sealants or restorations were regarded as sound. Moreover, the distinction of noncavitated and cavitated lesions was not clear as the CAST codes 3 and 4 were employed to define lesions in enamel and dentine, based on the surface discoloration only, with or without the surface breakdown. The CAST classification is not designed to assess activity status of the lesions. Thus, despite certain similarities between the studied populations (similar geographical and cultural-economical backgrounds and the investigation period), direct comparison of the data obtained from these surveys is not fair.

In the present study, the Nyvad criteria for the clinical assessment of caries lesions according to their severity and activity status were used. These criteria have been shown to be reliable and valid when used by trained examiners [8,13,29,30,31]. In the present study, the criteria for the first time were employed in a national survey. The examiner was extensively trained and calibrated by the experienced examiner, one of the developers of the Nyvad clinical classification of the caries lesions. The data collected during the survey provided an important information about the unmet treatment needs in Lithuanian children. Given the fact that the majority of the detected caries lesions (either noncavitated or cavitated) were recorded as active, the obtained data should be a call for action in order to define an appropriate management strategy in this population. Although the present study sample of individuals attending pre-school institutions was not truly representative of all Lithuanian 5–6-year-old children, and certain limitations of the multistage cluster sampling should be acknowledged, it was sufficient to demonstrate the existing dental care problems in a significant part of the population of this age.

The evidence about the efficacy of population-based interventions on oral health in general is not consistent [32], although, in child populations, a high-profile of community, in office and individual preventive measures have been shown to be effective in a number of countries [33]. Unfortunately, the dental health services in Lithuania (both private and public) are mainly focused on restorative treatments. Moreover, the public oral health care services in educational institutions hardly exist. This means that control of the progression of dental caries is essentially a matter of individual understanding and personal efforts of the Lithuanian residents. Therefore, the most likely way to address the problem in this population would be an individual approach, supported professionally and based on caries risk assessments, careful evaluation of the actual treatment need and patient counseling in order to promote beneficial behavior [6,32,34,35,36].

## 5. Conclusions

The obtained data indicate high-treatment needs in the erupting first permanent molars of Lithuanian 5–6-year-old children attending pre-school institutions and imply that caries management in this population should primarily focus on improved biofilm removal, accompanied with the regular use of fluoridated toothpaste.

## Figures and Tables

**Table 1:** Dental caries profile in the first permanent molars among 5- and 6-year-old Lithuanian children.

Age Group, *n*	DF-S (WHO)	DF-S (Nyvad)	Decayed Surfaces (Nyvad Criteria)					Filled Surfaces
			Total	Active, Noncavitated	Active, Cavitated	Inactive, Noncavitated	Inactive, Cavitated	
	M (SD), Median (IQR 25%–75%)							
5-year-olds	0.33 (1.02)	1.01 (2.17)	1.01 (2.17)	0.63 (1.54)	0.33 (1.02)	0.05 (0.45)	0	0
n = 79	0 (0–0.0) *	0 (0–0.0) *	0 (0–0.0) *	0 (0–0.0)	0 (0–0.0) *	0 (0–0.0) *		
6-year-olds	0.81 (1.48)	1.95 (3.05)	1.84 (2.93)	0.95 (2.06)	0.67 (0.28)	0.19 (0.90)	0.03 (0.27)	0.11 (0.7)
n = 374	0 (0–1.0)	0 (0–3.0)	0 (0–3.0)	0 (0–1.0)	0 (0–1.0)	0 (0–0.0)	0 (0–0.0)	0 (0–0.0) *
*p* value	<0.001	0.002	0.004	0.12	0.01	0.04	0.34	0.003
5–6-year-olds	0.73 (1.42)	1.79 (2.93)	1.7 (2.83)	0.89 (1.98)	0.61 (1.24)	0.17 (0.84)	0.02 (0.24)	0.09 (0.64)
n = 453	0 (0–1.0)	0 (0–3.0)	0 (0–3.0)	0 (0–1.0)	0 (0–1.0)	0 (0–0.0)	0 (0–0.0)	0 (0–0.0)

* *p* < 0.05, statistically significant difference between 5- and 6-year-olds, Mann-Whitney U test. DF-S, decayed and filled surfaces; WHO, World Health Organization; M (SD), mean (standard deviation); IQR, interquartile range.

**Table 2:** Dental caries profiles in occlusal surfaces of the first permanent molars of 5–6-year-old Lithuanian children.

Age Group, *n*	Decayed Surfaces (Nyvad Criteria)				
	Active, Noncavitated	Active, Cavitated	Inactive, Noncavitated	Inactive, Cavitated	Filled Surfaces
	M (SD), Median (IQR 25–75%), Prevalence N (%)				
5-year-olds *n* = 79	0.35 (1.24)	0.19 (0.68)	0.05 (0.45)	0	0
	0 (0–0.0)	0 (0–0.0)	0 (0–0.0)		
	11 (14%)	7 (9%) *	1 (1%)		
6-year-olds *n* = 374	0.66 (1.85)	0.51 (1.0)	0.14 (0.63)	0.03 (0.27)	0.07 (0.34)
	0 (0–1.0)	0 (0–1.0)	0 (0–0.0)	0 (0–0.0)	0 (0–0.0)
	65 (17%)	94 (25%) *	24 (6%)	5 (1%)	17 (5%)
*p* value	0.361/0.455	0.002/0.002	0.072/0.068	0.302/0.592	0.054/0.053
5–6-year-olds *n* = 453	0.61 (1.76)	0.45 (0.97)	0.13 (0.6)	0.02 (0.24)	0.06 (0.34)
	0 (0–0.0)	0 (0–0.0)	0 (0–0.0)	0 (0–0.0)	0 (0–0.0)
	76 (17%)	101 (22%)	25 (6%)	5 (1%)	17 (4%)

* *p* < 0.05, statistically significant difference between 5- and 6-year-olds, Mann-Whitney U (M/N) and χ^2^ tests.

**Table 3:** Distribution of active caries lesions in the first permanent molars and odds ratio estimates for an association between the prevalence of active caries lesions and the dental plaque levels, as well as the self- reported toothbrushing habits among 5- and 6-year-old children.

Age, Years	Active Caries (Noncavitated and Cavitated Lesions)		
		*n* (%) of Individuals with Active Lesions	OR (95% CI)
	Dental plaque (plaque scores)		
5–6	No plaque (0+1), n = 224	34 (15)	1 7.36 (4.70–11.52)
	Plaque present (2+3), n = 230	131 (57)	
	*p* value *	<0.001	
5	No plaque (0+1), n = 35	4 (11)	1 4.01 (1.19–13.49)
	Plaque present (2+3), n = 44	15 (34)	
	*p* value	0.019	
6	No plaque (0+1), n = 188	30 (16)	1 8.73 (5.35–14.25)
	Plaque present (2+3), n = 186	116 (62)	
	*p* value	<0.001	
	Self-reported toothbrushing frequency		
5–6	1–2 times per day, n = 360	103 (29)	1 4.99 (3.063–8.13)
	Irregularly/never, n = 93	62 (67)	
	*p* value	<0.001	
5	1–2 times per day, n = 58	8 (14)	1 6.88 (2.21–21.41)
	Irregularly/never, n = 21	11 (52)	
	*p* value	<0.001	
6	1–2 times per day, n = 302	95 (32)	1 5.29 (3.01–9.29)
	Irregularly/never, n = 72	51 (71)	
	*p* value	<0.001	

* χ^2^ test. OR, odds ratio; CI, confidence interval.

**Table 4:** Multivariate logistic regression of the predictors of a high caries experience* in the permanent teeth of Lithuanian 5–6-year-olds.

Predictor	High DF-S, Odds Ratio (95% CI)
High % dmfs *	4.2 (2.7–6.8)
Age, years	2.7 (1.3–5.4)
Self-reported tooth brushing frequency	2.5 (1.5–4.3)
Nagelkerke R Square = 0.3; Overall Percentage = 81.2	

* A high caries experience in primary and in permanent dentition was defined as belonging to the upper tertile (dmfs > 21; DF-S > 2) of the distribution of dmfs and of DF-S values, respectively.
